# Third Annual Meeting of the Alumni Association of the Ohio College of Dental Surgery

**Published:** 1881-03

**Authors:** E. G. Betty


### THIRD ANNUAL MEETING
OF THE
### ALUMNI ASSOCIATION
OF THE
### OHIO COLLEGE OF DENTAL SURGERY.

The meeting of this body was called to order by President J. Taft, at 12 o'clock M., Wednesday, March 3d, 1881. The minutes of last year's meeting were read and approved. Treasurer N. S. Hoff made his annual report, which was accepted. The Executive Committee reported that arrangements were completed for a banquet at the Florentine Hotel.

The President was empowered to appoint a Committee of five to collect all possible data respecting the history of the College and its graduates, their report to be submitted to the Association at its meeting in 1882. The Committee is as follows: Drs. N. S. Hoff, Wm. D. Kempton, E. G. Betty, C. I. Keely and Geo. Watt.

On motion of Dr. H. M. Reid, the dues for this year were remitted, as there was money enough in the treasury to liquidate current expenses.

After the Commencement Exercises on Thursday evening, the 4th inst., the Association and its guests met at the Florentine Hotel, when the following officers for 1882 were elected:

| | | | |
|---|---|---|---|
| President, | G. W. Keely, | Class, | 1853. |
| Vice-President, | H. A. Smith, | " | 1859. |
| Secretary, | E. G. Betty, | " | 1876. |
| Treasurer, | N. S. Hoff, | " | 1876. |

The party then adjourned to the dining-hall, and sat down to the following bill of fare:

Oysters on half shell; Soup, Mock Turtle; Fish, Mackinaw Trout, Anchovy Sauce; Entrees, Fillet of Beef, Mushrooms, Baked Mashed Potatoes, Chicken Croquette, French Peas, Larded Sweet Breads, with Toast; Roast, Saddle Venison, with Currant Jelly; Cold Dishes, Chicken Salad, Tongue, Ham, Cheese, Celery,

Pickles; Fruits, Oranges, Apples, Bananas; Cake, Large Cake, Assorted Cakes. Vanilla Ice Cream. Coffee.

TOASTS.

1st.— *Our Alma Mater.* - - DR. G. W. KEELY.
2d. — *The Founders of the Ohio College of Dental Surgery*, DR. JAS. TAYLOR.
3d. — *Chemistry the Hand-maid of Dental Science*, DR. J. S. CASSIDY.
4th.—*Dental Progress*, - - - - DR. J. TAFT.
5th.—*The Dental Society of Europe*, - - DR. J. R. CLAYTON.
6th.—*The Profession at Large*, - - - DR. J. W. JAY.

The following gentlemen were admitted to membership by signing the Constitution and paying initiation fee:

M. H. Fletcher, W. D. Kempton, C. W. Wardle and E. H. Rothe, of Cincinnati; H. D. Eggers, Louisville, Ky.; J. C. Heron, Mt. Carmel, Ind.; B. P. Ingram, Moscow, O.; W. L. Hughes, Richmond, Ky., B. G. Rees, Winchester, Ky.; W. G. Price, Evansville, Ind.; C. S. Archer, Evansville, Ind.; W. C. Nesbitt, Owingsville, Ky.

Adjourned to meet again during the first week in March, 1882.

E. G. BETTY, *Secretary.*

